# Effect of intravitreal injection of anti-interleukin (IL)-6 antibody in experimental autoimmune uveitis in mice

**DOI:** 10.1186/s12348-024-00441-x

**Published:** 2024-11-04

**Authors:** Kristin Hösel, Büsra Chasan, Jan Tode, Stefan Rose-John, Johann Baptist Roider, Christoph Ehlken

**Affiliations:** 1 Department of Ophthalmology, UKSH Kiel, Kiel, Germany; 2 Department of Ophthalmology, MHH Hannover, Hannover, Germany; 3 Department of Biochemistry, Klinik für Augenheilkunde, CAU Kiel, Haus B2, Arnold-Heller-Str. 3, 24105 Kiel, Germany

**Keywords:** Uveitis, anti-IL-6 antibody, Experimental autoimmune uveitis, Non-infectious uveitis, Intravitreal therapy

## Abstract

**Purpose:**

The aim of this study was to assess the functional and clinical impact of intravitreal administration of a neutralizing anti-IL-6 antibody in the treatment of experimental autoimmune uveitis (EAU) in mice.

**Methods:**

EAU was induced in 17 female B10.RIII mice by administering Inter-Photoreceptor-Binding-Protein (IRBP) in complete Freund’s adjuvant, followed by a boost with Pertussis toxin. Intravitreal injections of anti-Interleukin (IL)-6 antibody were administered on days 10, 13, and 16 after EAU induction (day 0) into the randomized treatment eye, with an isotype antibody similarly injected into the fellow control eye. Visual acuity was assessed using the optomotor reflex via OptoDrum, and clinical scoring was performed via fundus imaging (utilizing 6 EAU grades) in a single-blinded manner on days 0, 10, 13, 16, and 18.

**Results:**

Uveitis developed in all 17 mice. Significantly higher visual acuity was observed in treated eyes compared to control eyes on days 13, 16, and 18. The most pronounced effect was noted on days 16 and 18 (*p* < 0.001). On days 13, 16, and 18 the number of eyes with lower EAU-score was significantly higher in the treatment group, with the most notable effect observed on day 18 (*p* < 0.003).

**Conclusion:**

Intravitreal administration of anti-IL-6 treatment notably mitigates experimental autoimmune uveitis in mice, both functionally and clinically. Further investigations are warranted to assess the potential of intravitreal anti-IL-6 therapy as a treatment option for non-infectious uveitis in humans.

## Background

Uveitis is an inflammatory disease affecting the uvea, either due to infection or non-infectious causes. It is a prevalent reason for legal blindness in the Western world, with an incidence of over 50 cases per 100,000 person-years [1, 2]. Severe uveitis can lead to complications such as macular edema, secondary glaucoma, blood vessel occlusion, and retinal degeneration, resulting in vision deterioration. While infectious uveitis allows for pathogen identification and targeted treatment, the pathophysiology of non-infectious autoimmune uveitis is diverse and not completely understood.

Non-infectious uveitis is most commonly treated with Disease Modifying Antirheumatic Drugs (DMARDs), primarily corticosteroids and classical immunomodulating substances (cyclosporine, tacrolimus, azathioprine, methotrexate, mycophenolate, leflunomide) [3, 4]. Advances in understanding autoimmune uveitis pathomechanisms have led to the use of more specific treatments, including biological immune-modulating agents such as Tumor Necrosis Factor (TNF)-α blockers [5–7], Cluster of Differentiation (CD)20 antagonists [8], and Interleukin-6-Receptor (IL-6R) antibodies [9, 10].

However, systemic application of these treatments may come with side effects like increased risk of serious infections [11] and organ toxicity [12], necessitating an interdisciplinary approach. The intravitreal administration of drugs effectively minimizes systemic side effects while enhancing therapeutic efficacy by directly targeting the primary site of infection in uveitis. While this approach substantially reduces systemic adverse reactions, it can still lead to significant local side effects, which may require the discontinuation of treatment.

Intravitreal steroid treatment is effective for autoimmune uveitis but is associated with complications like cataracts and steroid response glaucoma [13]. Intravitreal TNF-α blockers have shown limited effectiveness and may even be harmful to the eye [14]. An ideal intravitreal agent for intermediate and posterior autoimmune uveitis treatment would be both effective and have minimal side effects.

The prevailing concept of autoimmune uveitis suggests that it arises from the breakdown of the eye’s immune privilege, where under normal conditions, antigen presentation and T-cell response are inhibited [15, 16]. In uveitis, autoantigenic T-cells breach the blood-retina barrier, activating antigen-presenting cells and triggering an invasion of macrophages, CD4-positive (CD4+) T-cells, neutrophils, and CD8-positive (CD8+) T-cells [17]. Animal models indicate that the main effector cells are Interferon (IFN)γ -producing T helper (h)1-cells and IL-17-producing Th17-cells [15, 18, 19]. Various cytokines, including Interleukin-6 (IL-6), play a significant role in the inflammatory cascade of uveitis. IL-6 promotes the differentiation of Th1 to Th17 cells and inhibits physiological intraocular T-cell apoptosis, exacerbating inflammation [20–22]. Systemic inhibition of IL-6 has demonstrated efficacy in alleviating uveitis in both animal models [23, 24] and humans [9, 10]. However, IL-6 also plays a crucial role in metabolism, cell protection, development, and memory [25–27]. Systemic blockage of IL-6 may carry the risk of various adverse events, as documented in literature [28].

Intravitreal anti-IL-6 activity treatment for macular edema in uveitis patients is already under investigation (NCT05642325). Results of this multicenter, randomized, double-blind, placebo-controlled Phase III study are expected in 2025. However, up to our knowledge, there is only one preclinical study published to analyze the effect of intravitreally applied anti-IL-6 therapy in mice. Tode et al. conducted a pilot study with a low number of mice to evaluate the effect of an intravitreal anti-IL-6 antibody on experimental autoimmune uveitis (EAU) [29].

The present prospective randomized intra-individually controlled single-blinded study was conducted to confirm these findings in a larger number of subjects and to further explore the potential of intravitreal anti-IL-6 application for treating EAU.

## Main article

### Materials and methods

#### Animals

B10.RIII mice purchased from the Jackson Laboratories (The Jackson Laboratory, Bar Harbor, Maine, USA) were bred and maintained in the university animal care facility under specific-pathogen free conditions. All animals were housed under standard conditions (12 h daylight, 22 ± 2 °C, free access to water and rodent food). Only female mice older than 8 weeks were used in this study. Experiments were conducted in accordance to the EU directive 2010/63/EU for animal experiments. Procedures and experimentation were approved by the animal ethics and welfare committee (located at the ministry of energy transition, agriculture, environment and rural areas in Schleswig-Holstein, Number V 242-23935/2019 (52 − 5/19))) acting based on German federal and European law and adhered to the ARVO Statement for the Use of Animals in Ophthalmic and Vision Research.

#### Induction of experimental autoimmune uveitis (EAU)

EAU was induced following a protocol published by Caspi and colleagues [30]. An emulsion of 100 µl Interphotoreceptor retinoid-binding protein (IRBP 161–180, Hölzel Diagnostika, Cologne, Germany) and 100 ml complete Freund’s adjuvant (Sigma-Aldrich, Steinheim, Germany) enriched with 2.5 mg/ml Mycobacterium tuberculosis (desiccated strain H37Ra, DifcoTM, BD BioSciences Research, Heidelberg, Germany) was injected subcutaneously. EAU was boosted by an intraperitoneal injection of 1 mg pertussis toxin (Sigma-Aldrich) in 100 ml saline at the day of immunization. The induction of EAU was carried out under deep anesthesia.

#### Animal anesthesia and funduscopy

The animals were anesthetized systemically by intraperitoneal injection of 0.05 mg/kg body weight Fentanyl (Braun, Melsungen, Germany), 5.0 mg/kg bodyweight Midazolam (Hameln Pharma, Hameln, Germany) and 0.5 mg/kg bodyweight Medetomidin (CP-Pharma, Bergdorf, Germany). For the examination of the fundus, the eyes were dilated with a combination of 0.025 mg/ml Phenylephrine and 0.05 g/ml Tropicamide (UKSH Pharmacy, Kiel, Germany). A moisturizing gel was applied to protect the eye. Funduscopy was performed with a Micron III camera (MICRON III, Phoenix Research Laboratories, Pleasanton, CA, USA). During the examination 3 photos (central, nasal and temporal retina) were taken. To evaluate the disease severity a grading system with a 0–4 scale was used, where higher values stand for more severe disease. The following parameters were evaluated: papilledema, engorged blood vessels, constricted blood vessels (“cuffing”), large confluent lesions, subretinal hemorrhages, and retinal detachment [30, 31].

For fluorescein angiography, 10% Fluorescein (Alcon Pharma, Freiburg, Germany) was injected intraperitoneally on the last day of examination (60 mg/kg bodyweight in 100 ml saline). Angiography images were taken (Micron III) and evaluated according to a grading system similar to the abovementioned clinical score (0 = normal findings, 0.5 = optic disc leakage, 1 = little vascular leakage, 2 = vascular leakage, tortuous vessels and sheathing, 3 = chorioretinal leakage and capillary dropout, 4 = vessel abruption, global leakage, scarring and atrophy).

#### Visual acuity testing

Visual acuity was measured with an OptoDrum (Striatech, Tübingen, Germany) by non-invasive observation of the optomotor reflex [32]. The mice were placed on an elevated platform in an arena of monitors which showed horizontally drifting stripe patterns of various spatial frequencies (measured at 99,7% contrast) at a drift velocity of 12°/second. Visual acuity was defined in cycles per degree (cpd). Spatial frequency of the pattern was reduced following a staircase until no optomotor reflex was triggered. Both eyes were measured separately and by an examinant blinded for the treatment. Animals were examined five times (day 0, 10, 13, 16 and 18) during this study.

#### Intravitreal injections

A total of 3 intravitreal injections per eye were conducted in a randomized manner. One eye received anti-IL-6 antibody (*clone*: MP5-20F3, In Vivo Abcam, Berlin, Germany) and the fellow eye received an isotype rat IgG1 antibody (ltra Leaf Purified Rat IgG1; Isotype: Ctrl; Lot: B241906; Clone RTK207; BioLegend, San Diego, Kalifornien, USA). The injections were administered 10, 13, and 16 days after immunization. For intravitreal injection a Microinjection Syringe Pump (UltraMicroPump3, WPI, Sarasota, Florida, USA) was used to inject the total volume of 2000 nl including 3.6 µg of anti-IL-6 antibody, respectively 3.6 µg of isotype antibody, in 2,000 nl PBS solution in a microliter syringe (NanoFil, WPI) with a 35 Gauge beveled tip needle (NanoFil, WPI). All intravitreal injections were done under deep anesthesia.

After the injection, conjunctiva and cornea were given a drop of Ofloxacin 3 mg/ml (Floxal, Bausch&Lomb, Berlin, Germany) to prevent infection.

### Statistical analysis

Statistical analysis was conducted using IBM SPSS Statistics software (version 28, Armonk, NY, USA). To assess the assumption of normal distribution of the data, the Shapiro-Wilk test was conducted. In cases where the data were not normally distributed, non-parametric tests were used as an alternative to parametric tests. To investigate differences in visual acuity and EAU scores over the course of treatment, the Friedman test was used for non-parametric data. To evaluate statistically significant differences between treated and control eyes the Wilcoxon signed-rank test was used for non-parametric data. A significance level of 5% was set for all statistical calculations, with p-values < 0.05 considered statistically significant.

## Results

EAU was induced and developed in 17 female B10.RIII mice. Overall, 17 eyes were treated with anti-IL6 antibody (referred to as treatment group) and 17 eyes were treated with isotype antibody (referred to as control group). Intravitreal injections were uneventful.

### Effect of anti-IL-6 on EAU score

**Fig. 1:**
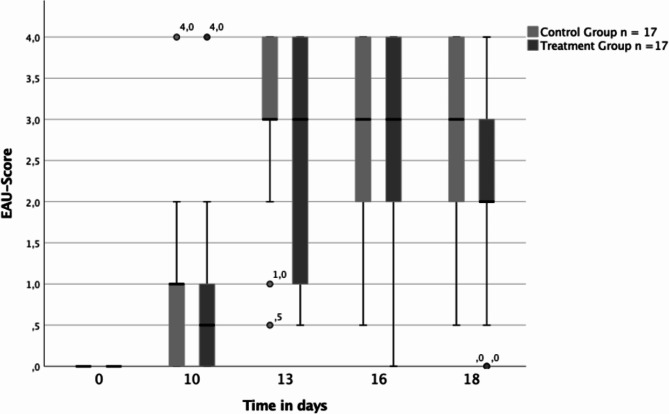
Boxplots illustrating EAU score for both the control group (light gray) and the treatment group (dark gray) over the examination days in *n* = 17 eyes each. The boxplots show the median EAU score, the 25th percentile, the 75th percentile, the interquartile range, as well as the minimum and maximum values. Outliers are also marked separately

As illustrated in Fig. 1, analysis of the median EAU score revealed no significant difference in both groups on all examination days. On day 10 after immunization, clinical assessment indicated that most eyes showed a low degree of EAU, with a median clinical score of 0,5 in the treatment group and 1 in the control group. Analysis at day 10 post-immunization revealed no significant difference (*p* = 0,339) between the treated eyes and their respective control eyes.

**Fig. 2:**
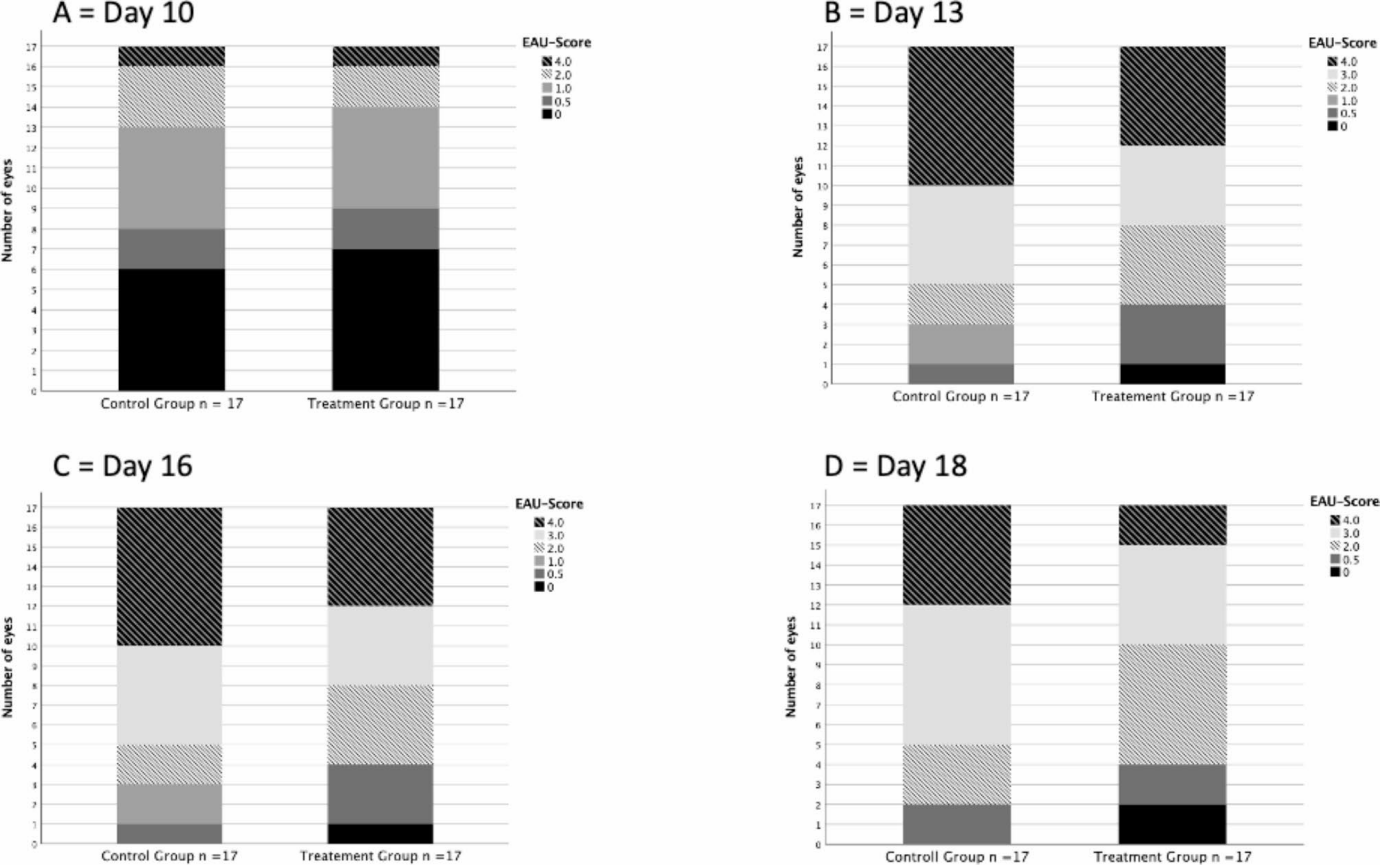
Bar chart depicting absolute frequency of each EAU score according to each group on examination days 10, 13, 16 and 18

However, as depicted in Fig. 2, on days 13, 16, and 18 the number of eyes with lower EAU-score was significantly higher in the treatment group, with the most notable effect observed on day 18.

By day 13 post-immunization, most eyes in both groups (76% of the control eyes (13/17) vs. 65% of the treated eyes (11/17)) had an EAU score ≥ 3 characterized by extensive chorioretinal infiltration, vascular sheathing, vessel abruption, retinal edema, and subretinal, intraretinal, or vitreal bleeding. The difference between the treated and control group was significant (*p* < 0.034).

On day 16 and 18 post-immunization, the difference between treated and control eyes was significant (day 16 *p* < 0.01, day 18 *p* < 0.003). On day 16, 12 of 17 eyes (71%) in the control group still had a score of ≥ 3, while only 9 of 17 eyes (53%) in the treatment group had a score of ≥ 3. In the control group, all eyes had clinical uveitis findings, whereas in the treatment group, one eye (6%) already had an EAU score of 0.

On day 18, the number of eyes with severe to very severe uveitis in the control group remained at 12 of 17 eyes (71%), whereas only 7 of 17 treated eyes (41%) had a score ≥ 3. In addition, all control eyes continued to show signs of active inflammation, while 2 of 17 treated eyes (12%) no longer showed signs of active inflammation.

Angiographic scoring supported the clinical findings. Angiography was conducted on day 18. The median angiography score for eyes treated with anti-IL-6 was 2 (ranging from 0 to 4), while control eyes scored 3 (ranging from 0.5 to 4). This difference was statistically significant (*p* < 0.013).

**Fig. 3:**
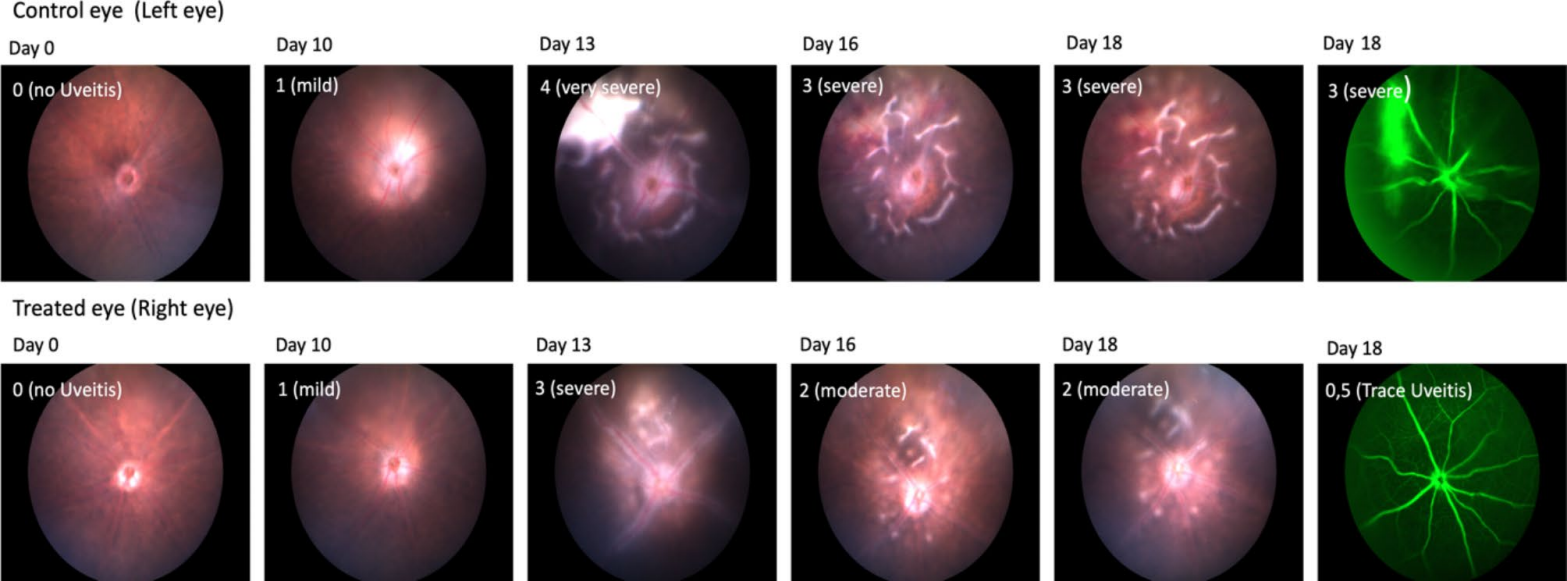
Funduscopy and fluorescein angiography images of a single mouse as an example. Upper row: findings of the control eye treated with the isotype antibody. Bottom row: findings of the eye treated with anti-IL-6 antibody. The days of the study are arranged from left to right. The EAU score is indicated in the upper right corner of each image

Exemplary depicted in Fig. 3, the course of EAU was more severe in control eyes than in treated eyes an anti-Il-6 led to a more significant improvement during the therapy compared to the control group. On day 18, fewer treated eyes had disease activity versus control eyes.

### Effect of anti-IL-6 on visual acuity

**Fig. 4:**
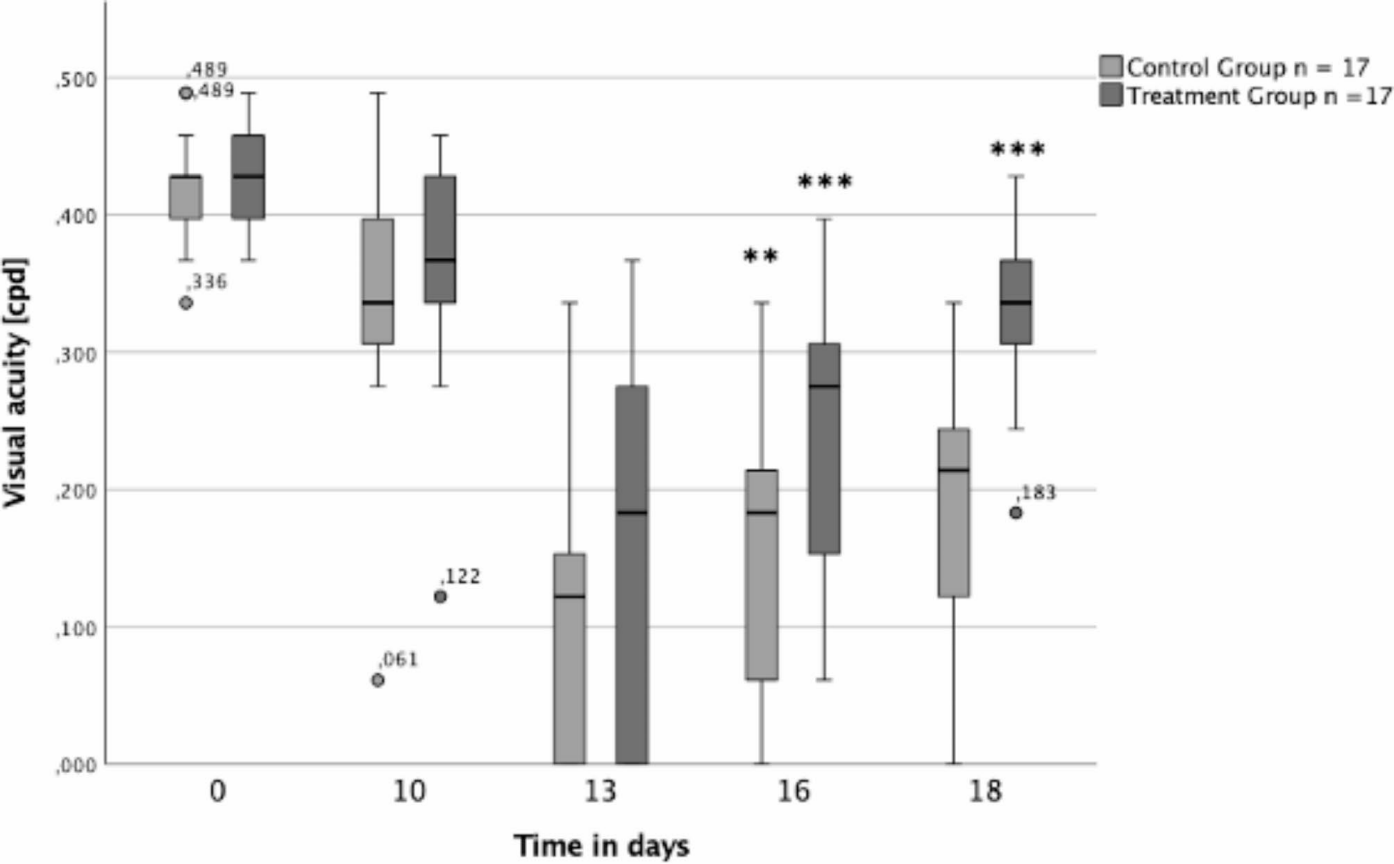
Boxplots illustrating visual acuity for both the control group (light gray) and the treatment group (dark gray) over the examination days in *n* = 17 animals. The Boxplots show the median visual acuity, the 25th percentile, the 75th percentile, the interquartile range, as well as the minimum and maximum values. Outliers are also marked separately. *p < 0.05, **p < 0.01, *** *p* < 0.001

At the beginning both groups had a median baseline visual acuity of 0.428 cpd (Fig. 4). After EAU induction an initial decline in median visual acuity was observed in each group with the lowest value on day 13 (0.122 cpd in control group versus 0.183 cpd in treatment group; *p* = 0.02). Median visual acuity improved afterwards in both arms on days 16 (0.184 cpd in control group versus 0.275 cpd in treatment group; *p* < 0.001)) and 18 (0.214 cpd in control group versus 0.336 cpd in treatment group; *p* < 0.001)). The administration of the anti-IL-6 antibody limited the decline in visual acuity following the initial injection and also led to a more significant improvement during the therapy compared to the control group.

**Fig. 5:**
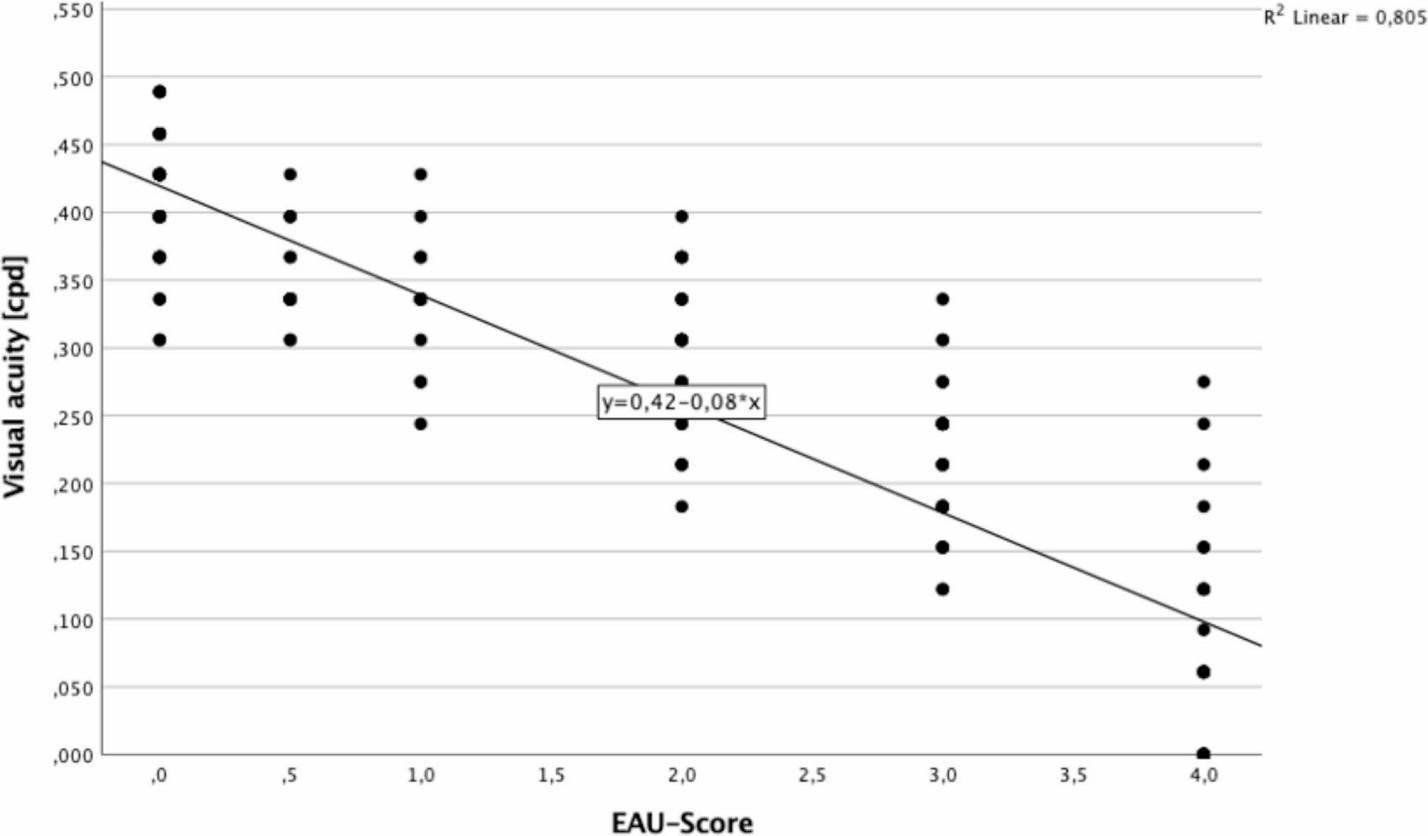
Determining visual acuity based on the EAU score. Representation of the regression line and the regression equation; the points depict the measured visual acuity values in cpd at their respective EAU scores

As shown in Fig. 5, visual acuity is associated and negatively correlated with the EAU score and correlates negatively with it (*p* < 0.001). Therefore, an initial decrease in visual acuity up to day 13 was accompanied by an increase in EAU score. As visual acuity increased, a decrease in EAU score was observed.

## Discussion

### Study protocol

To our knowledge, this is the second study to evaluate the effect of intravitreal injection of anti-IL-6 on EAU in mice. We conducted this study based on the preliminary study of Tode et al. [29] with slightly different characteristics: our study involved a larger number of mice, the injections were administered three times instead of twice and the control antibody used was an isotype rat antibody. Moreover, in this study setup, functional in addition to morphological parameters were used.

The progression of EAU with the administration of anti-IL-6 was studied in 17 female mice of the B10.RIII strain over an 18-day period. The B10.RIII strain is known to be the most susceptible strain for EAU development and is suitable for exploring new therapeutic approaches for non-infectious posterior uveitis in humans [33, 34]. The course of EAU during this study period corresponds with findings in the literature stating that EAU typically develops in the first week, reaches an inflammation peak towards the end of the second week, and then shows improvement after three weeks [30]. More than half of all mice showed severe to very severe uveitis.

In contrast to the pilot project, the injections in this study were performed at a later time point and were conducted three times in total. The first injection was administered on day 10, with subsequent injections at three-day intervals until day 16. This decision was made in consideration of the fact that EAU usually develops in the first week with apparent clinical symptoms after day 7. The data gathered here demonstrate that antibody administration at a later stage in the acute phase of inflammation also has a significantly positive impact on the clinical and functional course of EAU.

Currently, there is a lack of information regarding the stability and duration of the biological activity of the anti-IL-6 antibody in the mouse eye. Unlike Vascular Endothelial Growth Factor (VEGF) -inhibitors used intravitreally for treating exudative age-related macular degeneration, which have a short half-life in the eye of just a few days [35], no similar data exists for the anti-IL-6 antibody. Therefore, the injection intervals (three-day interval) and dosage (3.6 µg) in this study were chosen based on the pilot study done by Tode et al. (2017).

As a control antibody a murine IgG1 isotype antibody from the rat was used. Isotype antibodies are structurally similar to therapeutic antibodies, the main difference being that there are no binding sites for the target molecule (IL-6) in the Fab region. This feature gives isotype antibodies the advantage of serving as negative controls while attenuating potential immunological responses originating from non-specific binding sites such as Fc receptors. The outcomes from the control eyes were similar to the control eyes of the pilot study where PBS was injected. This indicated that the isotype antibody did not affect the natural course of EAU in terms of clinical evaluations. As a result, the isotype antibody proves to be a suitable negative control for intravitreal applications, distinguishing itself from other control agents like PBS or Saline by preventing immunologically mediated reactions caused by non-specific antibody binding.

Non-infectious uveitis is often treated with systemic drugs [36]. Among those, Tocilizumab, a recombinant humanized anti-IL-6 receptor antibody, has been shown to be effective [28, 37]. However, intravitreal injections would offer the advantage of achieving a higher drug concentration in the eye while reducing the incidence of systemic side effects [38].

The EAU serves as a model for autoimmune diseases induced systemically. T-cells become activated and primed in the periphery, subsequently migrating to the eye and initiating an autoimmune inflammatory cascade within the normally immune-privileged eye structures [15]. As a result, many EAU treatment approaches involve the systemic administration of anti-inflammatory or immune-modulating agents [39]. The local action of intravitreally applied anti-IL-6 is anticipated, focusing on intraocular inflammatory processes. Nevertheless, experiences with intraocular anti-VEGF treatment indicate that intravitreally administered antibodies may minimally breach the blood-retina barrier, appearing in peripheral blood [31, 40]. Consequently, a marginal systemic effect of intravitreally injected anti-IL-6 cannot be completely dismissed. However, if the primary effect were systemic, a decrease in uveitis activity would be expected in both the treated and control eyes. In the current study, 71% of control eyes exhibited grade 3 or 4 uveitis, whereas only 41% of treated eyes in the same individuals had uveitis scores of 3 and 4. Thus, we propose that the systemic impact of intravitreally administered anti-IL-6 has no significant influence on uveitis in the fellow eye. Even if there were systemic effects, the intraocular injections, initiated 10 days after EAU induction, would not affect the initial stages of EAU development based on the temporal sequence of events.

Besides clinical uveitis evaluation via funduscopy and fluorescein angiography, we additionally chose to measure visual acuity with the OptoDrum system on each examination day. This decision was based on the recognition that clinical observations of the eyes only cannot provide a comprehensive picture of anti-IL-6 therapy on EAU.

It proved to be a simple, rapid, and objective tool to assess functional limitations in awake and untrained mice [41]. As a result, the assessment of visual acuity not only confirmed the clinical efficacy of the anti-IL-6 antibody but also demonstrated its functional benefits.

### Impact of intravitreal anti-IL-6 treatment on EAU

The results generated in this study confirm the results of the mentioned pilot study and indicate that triple intravitreal administration of an anti-IL-6 antibody during the acute phase of EAU is both functionally and clinically superior to isotype antibody administration.

Measurements of visual acuity at day 0 in both groups ranged from 0.4 cycles per degree (cpd) to 0.6 cpd, which is comparable to values documented in previous studies for visual acuity in healthy mice [42]. Following the initial antibody treatment on day 10, both groups experienced a reduction in visual acuity, reaching its peak of inflammation on day 13. Nevertheless, the findings demonstrate that the administration of the anti-IL-6 antibody resulted in a less significant decline in median visual acuity within the treatment group compared to the control group, leading to a lower level of visual loss. Moreover, visual acuity showed a more notable improvement over time in the treatment group. Consequently, intravitreal anti-IL-6 treatment appears to be a promising approach for effectively targeting and managing inflammation, thereby enhancing the quality of life.

The EAU score data showed a statistically significant superiority for anti-IL-6 therapy in terms of reduction of inflammatory activity. On days 13, 16, and 18 the number of eyes with lower EAU-score was significantly higher in the treatment group. At the same time, the additional measurement of visual acuity demonstrated that the visual impairment of the treatment group was significantly lower than in the control group. The combination of visual acuity measurement and EAU score determination was shown to be beneficial in determining the degree of inflammation and eliciting functional impact. Angiography results supported this observation and showed a lower severity of vascular involvement in the treatment group.

### Limitations and future prospects

The results of this study show that the intravitreal anit-IL-6 therapy is a promising therapeutic approach for the treatment of non-inflammatory uveitis.

A limitation of our study is some ambiguity concerning the timing of injections and the dosing. Future studies should aim to prevent or ideally eliminate disease activity. The long-term goal should be the complete recovery of the disease. Thus, optimal dosage and intervals of treatment should be examined.

The dosage used in the pilot project and in this study has already shown a significant effect. It may be of interest to increase the dosage as this would potentially increase the effectiveness of the antibody. An alternative approach could be in the form of a boost therapy on day 10 to mitigate the peak EAU on day 13 and thus have a positive impact on the course of the disease.

Optimization of the treatment regimen will also require adjustment of the intervals between the injections. As only one preclinical study has investigated intravitreal administration of an anti-IL-6 antibody, the total number of injections required for a sustained therapeutic effect remains unclear.

The isotype antibody used in this study proved to be a reliable negative control. One way to assess the efficacy of the anti-IL-6 antibody compared with an already established therapy could be to introduce a positive control. Corticosteroids could serve as an appropriate choice in this regard.

Future investigations should also assess anti-IL-6 and IL-6 blood levels in treated mice to confirm the absence of any systemic effects.

## Conclusion

The application of intravitreal anti-IL-6 treatment presents a promising therapeutic approach for addressing non-infectious uveitis. In this preclinical mouse model, our data illustrate that the administration of an anti-IL6 antibody diminishes inflammation indicators (as assessed by EAU score) while concurrently alleviating functional impairment (evaluated as visual acuity) resulting from EAU.

## Data Availability

No datasets were generated or analysed during the current study.

## Abbreviations

CD: Cluster of Differentiation
Cpd: Cycles per degree
DMARDS: Disease Modifying Antirheumatic Drugs
EAU: Experimental autoimmune uveitis
IL-6: Interleukin-6
IL-6R: Interleukin-6-Receptor
INF: Interferon
IRBP: Inter-Photoreceptor-Binding-Protein
PBS: Phosphate-buffered saline
TNF: Tumor Necrosis Factor
VEGF: Vascular Endothelial Growth Factor
